# Long-term effects on swallowing and laryngeal function after treatment for severe COVID-19 disease in intensive care

**DOI:** 10.1007/s00405-024-08648-3

**Published:** 2024-04-20

**Authors:** Hans Dotevall, Lisa Tuomi, Ellen Lindell, Caterina Finizia

**Affiliations:** 1 Department of Otorhinolaryngology, Head and Neck Surgery, Institute of Clinical Sciences, Sahlgrenska Academy, University of Gothenburg, Gothenburg, Sweden; 2 Department of Otorhinolaryngology, Head and Neck Surgery, Sahlgrenska University Hospital, Region Västra Götaland, Gothenburg, Sweden; 3 Institute of Neuroscience and Physiology, Speech and Language Pathology Unit, Sahlgrenska Academy, University of Gothenburg, Gothenburg, Sweden; 4 Department of Research, Education and Innovation, Södra Älvsborgs Hospital, Region Västra Götaland, Borås, Sweden

**Keywords:** Deglutition disorders, Laryngeal function, Patient reported outcome measurements, Covid-19

## Abstract

**Purpose:**

This study aimed to assess swallowing and laryngeal function at long-term follow-up in patients treated for severe COVID-19 in the ICU.

**Methods:**

Thirty-six patients with severe COVID-19 were prospectively examined with fiberendoscopic evaluation of swallowing (FEES) about 6 and 12 months after ICU discharge. Comparison with initial FEES examinations during the time in hospital was performed in 17 patients. Analysis of swallowing function and laryngeal features was performed from video recordings. Twenty-five participants responded to Eating Assessment Tool, Voice Handicap Index, and the Hospital Anxiety and Depression Scale at follow-up.

**Results:**

Penetration to the laryngeal vestibule (PAS ≥ 3) was seen in 22% and silent aspiration (PAS = 8) in 11% of patients on at least one swallow at follow-up. Fourteen percent had obvious residue in the vallecula and/or pyriform sinuses after swallowing thick liquid or biscuits. Self-reported eating and swallowing difficulties were found in 40% of patients. Abnormal findings in the larynx were present in 53% at follow-up. Thirty-three percent had reduced or impaired vocal fold movement, of whom 22% had bilateral impaired abduction of the vocal folds. Possible anxiety and depression were found in 36% and 24% of responders, respectively.

**Conclusion:**

Although a majority of patients appear to regain normal swallowing function by 1 year after treatment for severe COVID-19, our results indicate that dysphagia, abnormal laryngeal function, and anxiety/depression may remain in a substantial proportion of patients. This suggests that swallowing and laryngeal function, and emotional symptoms, should be followed up systematically over time in this patient group.

## Introduction

Since March 2020, when the World Health Organization declared the novel coronavirus disease (COVID-19) caused by the severe acute respiratory syndrome coronavirus-2 (SARS-CoV-2) a pandemic health emergency, about 675 million cases have been confirmed worldwide including more than 6.8 million fatal cases until February 2023 [1]. About 2.7 million COVID-19 cases were reported in Sweden, a country with a population of about 10 million, including approximately 23,700 fatal cases and 10,000 cases treated in intensive care until February 2023 [2].

Previous studies have shown that oropharyngeal dysphagia is prevalent in COVID-19 patients treated in intensive care units (ICU). Dysphagia has been found in 31–93% of patients post extubation assessed with clinical bedside swallowing examinations [3–8]. A relatively large proportion of these patients exhibited signs of severe dysphagia [6, 7]. In a study using instrumental assessment of swallowing function with fiberendoscopic examination of swallowing (FEES) in a series of 25 patients with severe COVID-19 in our hospital, 96% had some degree of dysphagia during or shortly after their stay at the ICU [9]. The majority had moderate-to-severe dysphagia and 44% showed signs of silent aspiration to the trachea on at least one occasion.

At hospital discharge, 36% of patients with COVID-19 and treated in the ICU showed signs of dysphagia in the study by Gonzalez-Lindh et al*.* [4]. Mallart et al*.* [5] found normal feeding in 78% of previously intubated patients, while Regan et al*.* [7] observed that dysphagia persisted in 27% and 59%, respectively, at discharge from the hospital, even if swallowing function was improved in most cases.

There are to date a limited number of follow-up studies of swallowing function in COVID-19 patients treated in the ICU after hospital discharge. In published studies, the follow-up time is relatively short. Moreover, only clinical screening or assessment of swallowing function was used. There are to our knowledge no studies using instrumental methods to evaluate swallowing function. At follow-up 1–2 months after hospital discharge, Gonzalez-Lindh et al*.* [4] reported that 96% had no nutritional problems. In a group of patients who had undergone tracheotomy insertion and were subsequently decannulated, 83% had normal oral intake 2 months post-discharge [10]. Thirty percent of the patients reported subjectively impaired eating and swallow function on a self-assessment questionnaire in that study, however.

Laryngeal dysfunction and dysphonia also appear to be frequent in COVID-19 patients after treatment in the ICU [7, 9–12] as well as in mixed groups of patients post-COVID-19 [13–16]. Impaired vocal fold motion was seen in 50–100% of patients [9, 12, 14, 16]. Other common findings include laryngeal edema, subglottic stenosis, posterior glottic scar or stenosis, and laryngeal granuloma [9–14, 16]. Dysphonia and laryngeal abnormalities, such as vocal fold dysmotility, vocal fold edema, and laryngotracheal stenosis, have been found in patients treated for COVID-19 up to 8 months after hospital discharge in different studies [14–17]. The long-term consequences for these patients are still unknown.

The aim of this study was to evaluate long-term effects on oropharyngeal swallowing and laryngeal function after treatment of severe COVID-19 in intensive care using fiberendoscopic assessment of swallowing and laryngeal function and validated patient reported outcome measures (PROM).

## Materials and methods

### Patients

The participants in this observational, prospective study were patients treated in ICU at Sahlgrenska University Hospital (a tertiary hospital serving a population of about 1,000,000 in western Sweden) due to severe COVID-19 from the end of March 2020 to the beginning of June 2021. Inclusion criteria were polymerase chain reaction (PCR)-verified SARS-CoV-2 infection, treatment with mechanical ventilation in intensive care due to respiratory distress secondary to COVID-19, adult (≥ 18 years old), swallowing examination at follow-up about 6 and/or 12 months post discharge from the ICU, and informed consent to participate in the follow-up study. Patients with a previous history of dysphagia (e.g., due to neurologic or oncologic disease) and/or cognitive reduction, and patients with no informed consent were excluded. The participants were recruited from a cohort of patients with severe COVID-19 treated in the ICU in our hospital who were referred to the Department of Otorhinolaryngology for evaluation of swallowing function or tracheostomy.

Data of the total time in hospital, time in the ICU, time with intubation, time on ventilator, time with tracheostomy, comorbidities, and body mass index (BMI) were obtained from electronic medical records. The Adult Comorbidity Evaluation 27 scale [18], a chart-based validated comorbidity instrument, was used to describe the level of comorbidity in the patient cohort.

### Assessment

Assessment of swallowing function was performed by speech language pathologists using FEES. The speech language pathologists had more than 5 years of experience in dysphagia management. A fiberendoscopic examination of the larynx was included in the FEES protocol. The FEES was performed using a portable Xion EV-NC videofiberendoscope attached to a Xion Endoportable CFT-003 mobile workstation (Xion GmbH, Berlin, Germany) or a stationary Olympus ENF-VH videofiberendoscope attached to an Olympus Elite II OTV-S200 light source (Olympus Inc, Tokyo, Japan). The examinations were recorded digitally. Prior to the endoscopy, the nasal mucosa of the most patent nostril was decongested and anesthetized locally with a lidocaine 3.4%/naphazoline 0.02% solution using cotton attached to a thin feeding catheter (Unomedical Purifeed, CH 06), in order to reduce discomfort. Care was taken not to anesthetize the pharyngeal mucosa.

Personal protective equipment was used by the examiner and other staff in the room during the FEES, according to local hospital routine, in order to protect from possible decontamination by aerosol (N95/FFP3 face mask/respirator, covering gown, and gloves). No transmission of COVID-19 occurred due to the FEES examinations. The fiberscopes were cleaned and disinfected after each examination using an automated endoscope reprocessor (dishwasher) as recommended by the manufacturer.

Boluses with different consistencies and volumes colored with green coloring agent were presented using a standard protocol; thin non-carbonated liquid (corresponding to International Dysphagia Diet Standardisation Initiative, IDDSI level 0 [19]), mildly thick liquid (IDDSI level 2), extremely thick liquid/puree (IDDSI level 4), and biscuit (IDDSI level 6). In many cases, 3–5 ml carbonated water was given prior to the other types of boluses. The choice of boluses was made according to the clinical status and function of the patient, i.e., in cases with more severely impaired swallowing function at the time of the baseline FEES during hospital care, specific boluses that were considered unsafe or not necessary for the clinical assessment were avoided.

### PROMs

A Swedish version of the Eating Assessment Tool (S-EAT-10) [20, 21] was used for assessment of self-reported swallowing and eating function. This questionnaire includes ten items rated from 0 (no problems) to 4 (severe problems). Maximum score is 40 scale-points. Normative data indicate that a sum score of ≥ 3 can be regarded as abnormal [20, 21]. Using a cut-off score of ≥ 3, the sensitivity was shown to be 98.5% and specificity 94.1% in a comparison between a group of patients with dysphagia and a group of controls without dysphagia [21].

A short version of the Voice Handicap Index (VHI) [22], translated into Swedish [23], was used to assess self-perceived voice function. The shortened Swedish version of VHI (S-VHI-11) [23] includes the ten items from the original VHI-10 [22] in Swedish translation [24] with the addition of one item regarding throat discomfort. The maximum score is 44 scale-points on the S-VHI-11. In the original study by Rosen et al*.* [22], the median score was 1.0 and the mean score was 3.4 points (standard deviation, SD, ± 5.7) in a control group of 173 individuals. A score of ≥ 12 on the original VHI-10 [22] can be considered abnormal [25]. S-VHI-11 is validated in a Swedish cohort of voice patients and controls [23]. In a group of 36 healthy controls, the mean score was 2.1 points (SD ± 2.5) on the S-VHI-11. There is still no published normative cut-off score for the S-VHI-11. For the analysis, an estimated score of ≥ 13 points was used as cut-off for abnormality (i.e., 12 points linked to the original VHI-10 questionnaire plus 1 extra score point linked to the added item concerning throat symptoms).

The Hospital Anxiety and Depression Scale (HADS) [26] was used for assessment of mental health. HADS is a questionnaire with fourteen items, where seven items form a subscale reflecting self-perceived anxiety and the remaining seven items reflect self-perceived depression [26]. Each item is rated from 0 (best) to 3 (worst). On each subscale, a sum score of < 8 points is considered within normal range, a score of 8–10 suggests possible symptoms of emotional distress, whereas a score of ≥ 11 points indicates probable presence of a mood disorder [27].

All 36 participants also responded to specific questions concerning eating and swallowing: “Do you have difficulties with eating/drinking/swallowing?” (yes/no) and “Do eat and drink entirely by mouth?” (yes/no). All PROMs and study specific questions were administered in connection to the FEES.

### Analysis

Videos from the FEES examinations were analyzed by one medical doctor specialized in phoniatrics with more than thirty years of experience in the field of deglutition and laryngology and certified according to the European Society for Swallowing Disorders FEES accreditation program [28]. The video recordings were presented in a randomized order. Eighty-nine examinations were eligible for analysis. Sixteen percent of the videos were duplicated by randomization for analysis of intra-rater reliability. Another three FEES videos from patients examined 3 months post-ICU care were also duplicated, included in the randomization process, and added to the reliability analysis. Thus, a total of 89 original and 17 duplicated (19%) videos were evaluated. Duplicated videos with conflicting judgements of vocal fold motion or lesions were reassessed by two judges in order to reach a consensus judgement. Swallowing of carbonated water was excluded from the analysis since this was not given regularly.

The following variables were assessed:

1. Pooling of secretion in the pharynx and larynx before the first swallow was rated using the Murray Secretion Scale [29, 30], a four-grade scale where “1” refers to no or minimal secretion, and “4” to consistent secretion in the laryngeal vestibule.
2. Penetration to the laryngeal vestibule and aspiration during swallowing was assessed using the Penetration-Aspiration Scale (PAS) applied to FEES [31–33]. The PAS includes eight scale-steps where “1” denotes no material entering the airway and “8” indicates silent aspiration to the trachea, i.e., material enters the airway, passes below the vocal fold, and no effort is made to eject.
3. Residue in the vallecula and pyriform sinuses was rated according to the Yale Pharyngeal Residue Severity Scale [34, 35]. This is a five-grade scale where “1” is defined as no residue and “5” denotes residue filled up to the epiglottic rim or up to the aryepiglottic folds.
4. Movement of the vocal cords (normal, impaired/reduced, immobile).
5. Vocal cord lesions (e.g., contact granuloma, polyp).
6. Signs of inflammation in the larynx and pharynx (i.e., edema, erythema) (none, slight, moderate, severe).

### Statistics

For the main analysis, mean, median, range, and proportions of different ratings of impairment were calculated. Comparison between variables related to swallowing function at baseline during hospital care and follow-up as well as between the degree of residue in the vallecula and pyriform sinuses at follow-up were analyzed with the Wilcoxon signed-rank test. Correlation analyses were made with the Spearman’s rank correlation test. Intra-judge reliability was expressed as percent exact ratings, percent agreement within one scale step, and weighted kappa (κ) statistics [36]. In practice, a value of κ > 0.81 indicates very good agreement and κ of 0.61–0.80 good agreement, 0.41–0.60 moderated agreement, 0.21–0.40 fair agreement, and κ < 0.20 poor agreement [37]. SPSS Statistics version 29.0 for MacOS (IBM, New York, USA) and Microsoft Excel version 16.69.1 for Mac (Microsoft, Washington, USA) were used for the analyses.

### Ethical considerations

All participants gave written consent to participate in the study. The study was approved by the Swedish Ethical Review Authority (approval number 2020–03606). Authors had access to information to identify individual participants during and after data collection.

## Results

Thirty-six patients with severe COVID-19 infection treated in the ICU were included in the study. Thirty-two were male and four were female. Mean age was 64 years (range 43–80 years). The characteristics of the participants including comorbidities, time in hospital, and number of days in the ICU, on ventilator, with intubation, and with tracheostomy are shown in Table 1. Seventy-two percent (26/36) of patients were treated in the ICU for more than 15 days. All patients required invasive mechanical ventilation for a mean of 24 days (range 7–51 days) during their ICU stay. Seventy-two percent of patients (26/36) underwent tracheostomy. The total time in hospital was more than 30 days in 78% of patients (28/36). Pharmacologic treatment was utilized according to the routine at the ICU (i.e., medication with sedatives, cortisone, clonidine, and muscle relaxants when needed). Remdesivir or tocilizumab were given in a few cases.

**Table 1:** Characteristics of patients included in the study

Gender, n (%)	
Male	32 (89)
Female	4 (11)
Age, mean (range), years	64 (43–80)
BMI (kg/m^2^) at admission, n (%) ^a)^	
Under 25	5 (15)
25–30	13 (38)
31–40	12 (35)
40 +	4 (12)
BMI (kg/m^2^) at admission, mean (range)	32 (18–52)
Smoking, n (%)	
Non-smoker	22 (61)
Quit smoking > 12 months ago	9 (25)
Quit smoking < 12 months ago	3 (8)
Current smoker	1 (3)
No information	1 (3)
Comorbidities, n (%)	
None	5 (14)
Hypertension	23 (64)
Diabetes	17 (47)
Obstructive sleep apnea syndrome^b^	6 (17)
Stroke	0 (0)
Coronary heart disease	8 (22)
Adult Comorbidity Evaluation-27, n (%)	
Normal	4 (11)
Mild	20 (56)
Moderate	11 (30)
Severe	1 (3)
Days in hospital, mean (range)	52 (14–108)
Days in ICU, mean (range)	24 (7–51)
Days intubated, mean (range)^c^	10 (5–17)
Tracheostomy, n (%)	26 (72)
Days intubated before tracheostomy, mean (range)	11 (5–17)
Days with tracheostomy, mean (range)	26 (7–73)

*BMI* Body mass index, *ACE 27* Adult Comorbidity Evaluation 27 [18]

^a^Calculated on 34 patients because of missing BMI data for two patients

^b^According to medical records

^c^Including three patients reintubated in the ICU

An initial assessment of oropharyngeal swallowing function with FEES was performed during inpatient hospital care in 18 of the participants (baseline FEES). One video was lost due to technical problems, leaving 17 patients (43%) with baseline FEES available for analysis. The baseline FEES was done in connection with the relocation from the ICU to the hospital ward in eleven cases and at the end of the time in hospital in six cases. The remaining 19 patients were only evaluated at follow-up. Follow-up FEES were performed at a mean of 6 months after discharge from the ICU in 24 patients (range 5–8 months) and at a mean of 12 months in 32 patients (range 10–17 months). All participants were decannulated at the time of the follow-up investigations. Four participants who were assessed with FEES during hospital care declined follow-up at 12 months and where thus only examined at follow up at 6 months. There were no significant differences regarding FEES variables at the examination at 6 months between these four participants compared to the 20 participants who were examined with FEES at both 6 and 12 months.

### Secretions before swallowing

**Fig. 1:**
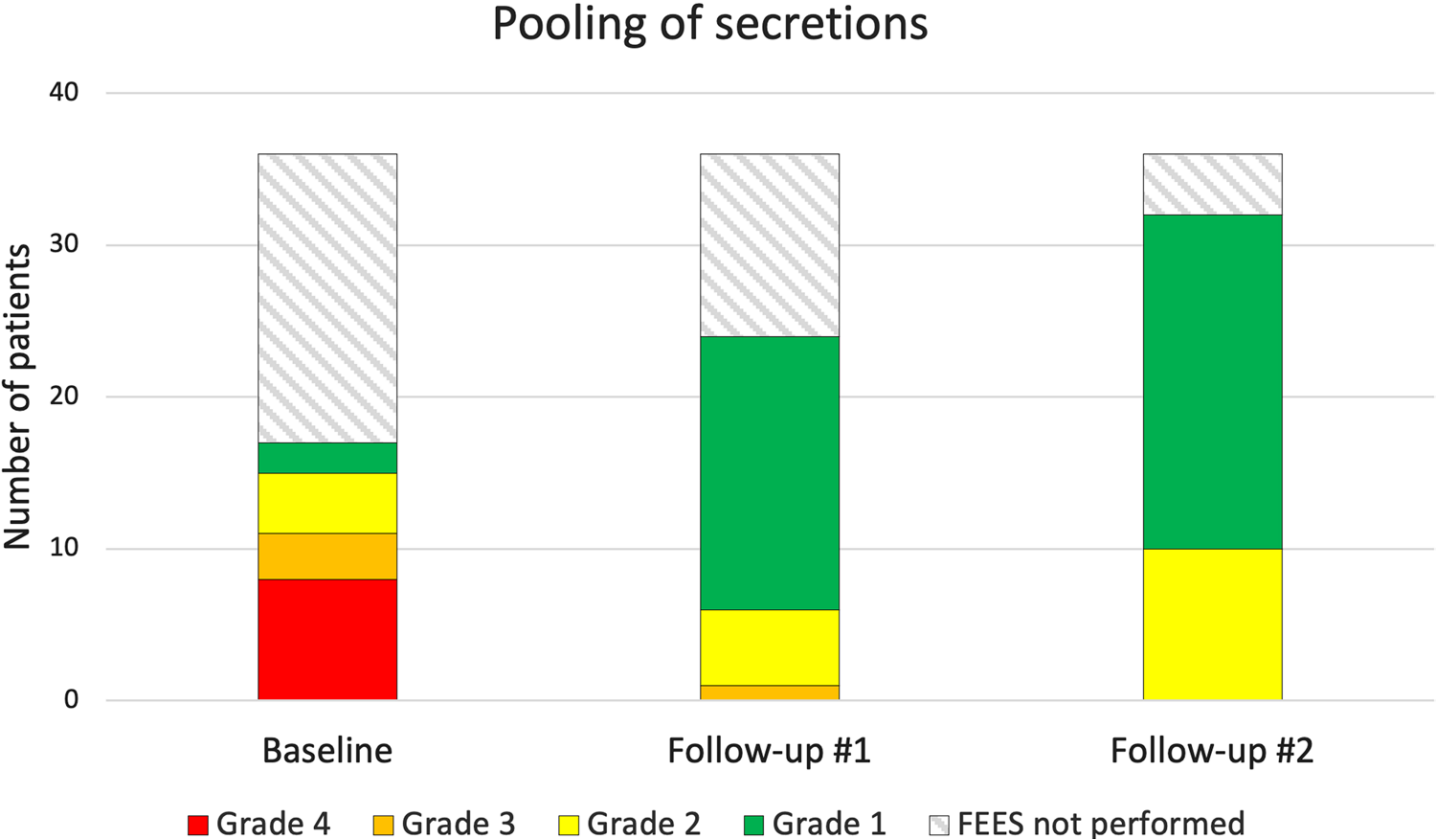
Pooling of secretions in the pharynx and larynx before the first swallow in COVID-19 patients treated in the intensive care unit (ICU). Ratings of secretion using the Murray Secretion Scale from the fiberendoscopic evaluation of swallowing (FEES) during hospital care (baseline), and at about six (follow-up #1) and twelve (follow-up #2) months after discharge from the ICU. Grade 1 refers to no or minimal secretion, and grade 4 to pooling of secretion in the laryngeal vestibule

In total, 26 of the 36 patients (72%) showed no or minimal secretions before the first swallow (grade 1) and 10 (24.4%) deeply pooled secretions in the pharynx (grade 2 according to the Murray Secretion Scale) at the last FEES 5–18 months after discharge from the ICU (Fig. 1). Pooling of secretion decreased significantly in the group of 17 patients with baseline examinations during the time in hospital in comparison to the follow-up FEES (median secretion score 3 [range 1–4] vs 1 [range 1–2], p < 0.001) (Table 2).

**Table 2:** Comparison between fiberendoscopic evaluation of swallowing (FEES) variables at the baseline and follow-up examinations

		Baseline FEES			Follow-up FEES			Statistics
Item	N*	Median	Mean	Range	Median	Mean	Range	P
Secretion	17	3.5	3	(1–4)	1	1.3	(1–2)	** < 0.001**
PAS								
Thick 5 ml	14	1	1.9	(1–8)	1	1	(1–1)	0.059
Thick 10 ml	11	1	2.9	(1–8)	1	1	(1–1)	0.066
Thin 3 ml	13	1	3.3	(1–8)	1	1.3	(1–3)	**0.034**
Thin 10 ml	12	3	3.6	(1–8)	1	1.8	(1–8)	**0.017**
Thin 20 ml	5	1	1.8	(1–3)	1	1.6	(1–4)	0.66
Biscuit	8	1	1.3	(1–3)	1	1	(1–1)	0.32
Residue vallecula								
Thick 5	14	3	2.6	(1–4)	2.5	2.4	(1–3)	0.37
Thick 10	11	2	2.5	(1–4)	3	2.6	(2–4)	0.94
Thin 3	13	2	2.3	(1–3)	2	2.2	(1–3)	0.48
Thin 10	12	3	2.6	(2–3)	2	2.3	(1–3)	0.10
Thin 20	5	3	2.6	(2–3)	2	2.2	(1–3)	0.16
Biscuit	8	2	2.3	(1–4)	1	1.5	(1–3)	0.16
Residue pyriform sinuses								
Thick 5	14	2.5	2.6	(1–4)	2	1.8	(1–3)	**0.013**
Thick 10	11	2	2.4	(1–4)	2	1.7	(1–3)	**0.008**
Thin 3	13	2	2.2	(1–3)	2	1.8	(1–3)	0.10
Thin 10	12	2	2.3	(2–3)	2	2	(1–3)	0.059
Thin 20	5	2	2.2	(2–3)	2	2	(1–3)	0.32
Biscuit	8	2	2	(1–4)	1	1.3	(1–2)	0.059

^*^In total 17 patients were examined with FEES during hospital care (baseline FEES). Only patients that were given the specific bolus types at the baseline FEES are included in the analysis. For the rest boluses that were considered unsafe were avoided

*PAS* Penetration-Aspiration Scale

Statistical comparison with the Wilcoxon signed rank test

### Penetration and aspiration score

**Fig. 2:**
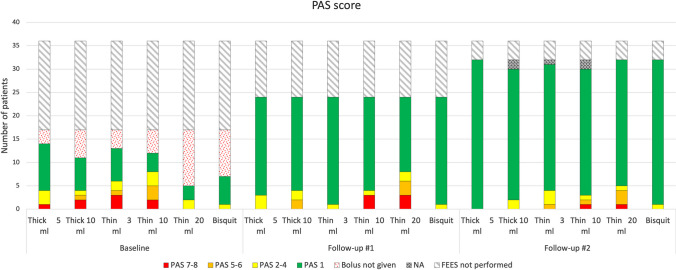
Penetration and aspiration scores (PAS) during swallowing in COVID-19 patients treated in the intensive care unit (ICU). PAS scores for different bolus consistencies (thick = mildly thick liquid, thin = thin liquid) at fiberendoscopic evaluation of swallowing during hospital care (baseline), at follow-up #1 (about 6 months after discharge), and at follow-up #2 (about 12 months after discharge) from the ICU. Grade 1 is no penetration or aspiration and grade 7–8 refers to aspiration to the trachea. The dotted red filling represents patients in whom the boluses were considered unsafe and not given due to high risk for aspiration. NA denotes ratings that were not possible to perform due to insufficient visibility on the video

Data of the depth of penetration and aspiration during the FEES according to the PAS analysis is summarized in Fig. 2. At follow-up five to eight months after discharge from the ICU, three of 24 individuals (12.5%) aspirated on larger volumes of thin liquid and another three patients exhibited penetration to the vocal folds on thin liquid (of whom two also had penetration to the vocal folds of 10 ml thick liquid). At follow-up ten to 17 months post-ICU discharge, one participant aspirated (3%) and three had penetration to the vocal folds (9%) on larger volumes of thin liquid. Three other patients (9%) had penetration into the laryngeal vestibule. In total seven patients (22%) had a PAS score of ≥ 3 on at least one bolus at the second follow-up FEES. Four patients (11%) showed signs of silent aspiration (PAS = 8) on at least one of the thin liquid boluses at follow-up. The PAS for 3 ml and 10 ml of thin liquid improved significantly at follow-up in the subjects who were examined with FEES during hospital care (Table 2). The change in PAS for 5 ml and 10 ml of thick liquid approached significance (Table 2).

### Residue after swallowing

**Fig. 3:**
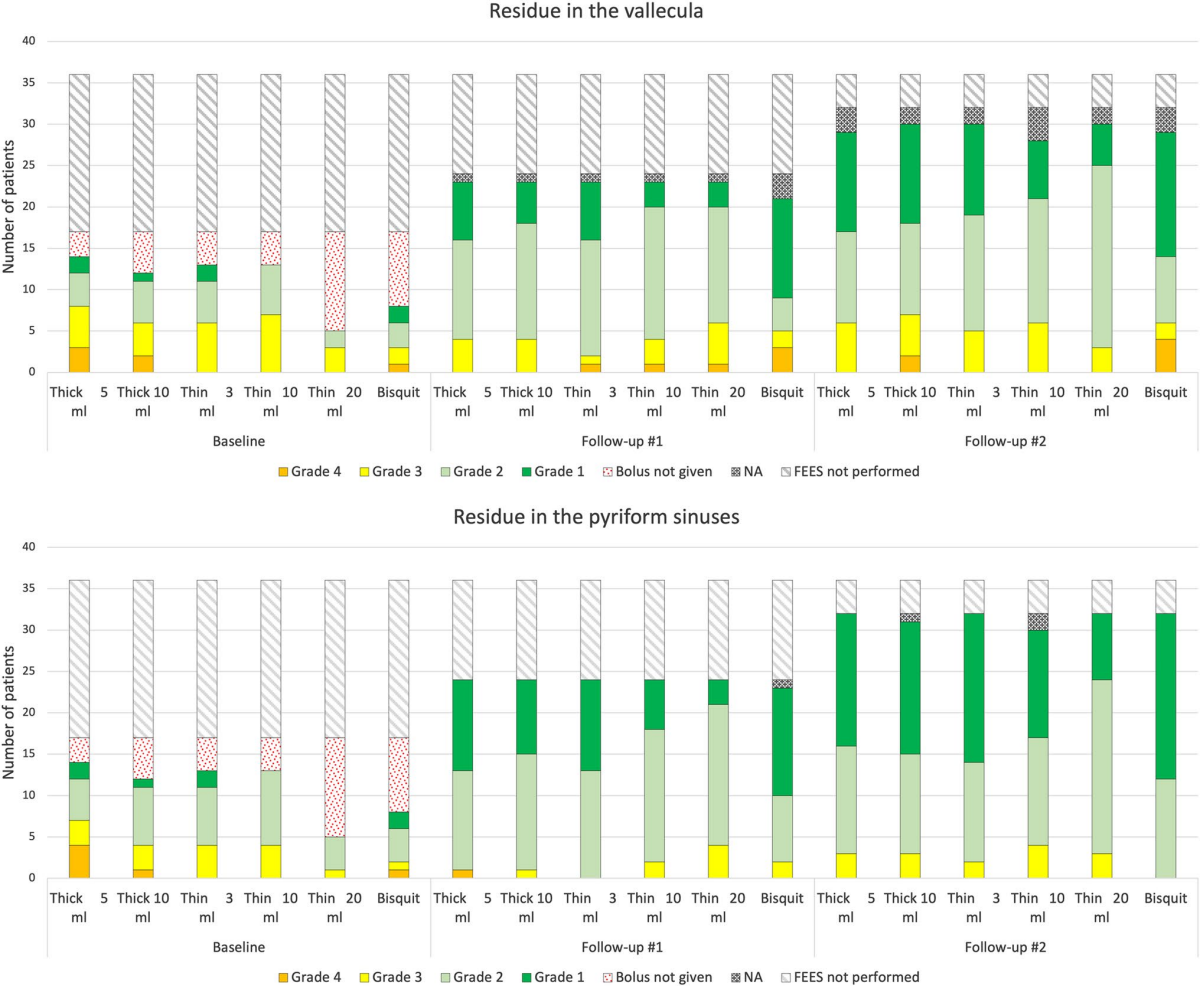
Residue in the vallecula (top) and pyriform (bottom) sinuses after swallowing in COVID-19 patients treated in the intensive care unit (ICU). Ratings of residue the Yale Pharyngeal Residue Scale at follow-up #1 (about 6 months) and at follow-up #2 (about 12 months) after discharge from the ICU. Grade 1 refers to “no residue” and grade 4 to “epiglottic ligament covered” or “up to half full”, respectively. The dotted red filling represents patients for whom the boluses were considered unsafe and not given due to high risk for aspiration. NA denotes ratings that were not possible to perform due to insufficient visibility on the video

Traces or small volumes of residue after swallowing (grade 2–3) were prevalent both in the vallecula and the pyriform sinuses at the follow-up FEES (Fig. 3). The estimated degree of residue was significantly higher in the vallecula than in the pyriform sinuses for all boluses at follow-up (p < 0.014), except for the 20 ml thin liquid bolus. Traces or mild degree of residue after swallowing (grade 2–3) was seen for at least one bolus consistency in all but three patients. A larger volume of residue (grade 4; epiglottic ligament covered) was noted in the vallecula after swallowing thick liquid or biscuit in five subjects (14%) at the second follow-up. The degree of residue in the vallecula did not change significantly between the baseline and follow-up FEES (Table 2). However, significant decrease of residue was seen in the pyriform sinuses for the 5 ml and 10 ml thick liquid boluses (p < 0.014) (Table 2).

### Laryngeal findings

Abnormal findings in the larynx were seen in 19 patients (53%) at follow-up. Vocal fold movement was reduced or impaired in twelve (33%) patients of whom eight (22%) had bilateral impaired abduction of the vocal folds (Table 3). Seven patients (20%) had contact granuloma or contact ulcer in the posterior larynx. Even though inspection of the trachea per se was not included in the examination protocol, tracheal stenosis was visible in two subjects (6%). One additional patient had synechia and stenosis of the posterior larynx, and, consequently, reduced mobility of the arytenoid cartilages as well as the vocal folds.

**Table 3:** Laryngeal findings at follow-up

Laryngeal findings	N* (%)
Vocal fold movement	
Normal	24 (67)
Right impaired/left normal	4 (11)
Impaired bilaterally	8 (22)
Vocal fold lesions	
Contact granuloma	6 (17)
Contact ulcer	1 (3)
Other lesions	
Posterior laryngeal stenosis	1 (3)
Visible tracheal stenosis	2 (6)
Vocal fold erythema	
None	30 (83)
Slight	4 (11)
Moderate	2 (6)
Vocal fold edema	
None	36 (100)

^*^Number of patients

In the group of 17 patients who were examined with FEES during their time in hospital care, the vocal fold mobility improved in seven (41%). Six (35%) had normal vocal fold movement and four (24%) patients had bilateral impaired abduction of the vocal folds both at baseline and follow-up. In two patients, contact granulomas had disappeared between the baseline and the follow-up examination. Four other patients, however, had developed contact granulomas at the time of the follow-up FEES. Two patients with signs of hyperkeratosis at the first FEES had normal laryngeal status at the last FEES. Six patients (17%) had some degree of erythema on the vocal folds. None had visible vocal cord edema on the last follow-up examination.

### PROMs

Twenty-five patients completed the S-EAT-10, S-VHI-11, and HADS questionnaires at the 6 and/or 12 months follow-ups. There were no statistical differences between PROM responders and non-responders on any of the study variables including patient characteristics (p > 0.05).

Self-reported eating and swallowing function assessed with S-EAT-10 was generally in the lower end of the scale (median 1 point; range 0–25). Ten of the 25 responders (40%) had a score of ≥ 3 points on the S-EAT-10 questionnaire (which was used as a cut-off score for dysphagia in the study by Möller et al*.* [22]) and five (20%) had a score of > 11 points, indicating a more pronounced impairment of eating and swallowing function.

Subjective voice function assessed with the S-VHI-11 questionnaire showed a similar pattern at follow-up (median 5 points; range 0–33). The majority (18/25; 72%) had a score of less than 13 points. Seven responders (28%) had a score of 13 points or more of whom two (8%) scored more than 20 points, indicating abnormality.

Three of the participants (12%) presented symptoms of possible anxiety (8–10 score points) and six (24%) presented symptoms indicating probable anxiety (≥ 11 score points) on the anxiety subscale of the HADS questionnaire at follow-up (median 5 points, range 0–17). On the depression subscale of the HADS, three (12%) had a score indicating possible depression (8–10 score points) and three (12%) others had a score indicated probable depression (≥ 11 score points) (median 5 points, range 0–14).

There were no obvious correlations between the results of the PROM questionnaires and the results of the FEES examination (Spearman rank correlation; p > 0.05). Eight of ten patients who scored ≥ 3 points on the S-EAT-10 questionnaire had normal PAS scores (PAS = 1) on the FEES and low scores for residue after swallow. One patient with a PAS score of 4 on one thin liquid bolus (i.e., penetration to the level of the vocal folds) had an S-EAT-10 score of 3 and another patient with a PAS score of 8 on thin liquid boluses (i.e., silent aspiration to the trachea) had a S-EAT-10 score of 12. Three patients with PAS scores between 5 and 7 (penetration to the level of the vocal cords or aspiration) on at least one bolus scored low on S-EAT-10 (score 0 to 3).

There appeared to be an association between abnormal laryngeal findings and higher scores on the S-VHI-11. Four of the seven patients with contact granuloma or contact ulcer scored above 10 points on the S-VHI-11. Three of the eight subjects with reduced vocal fold mobility and all three patients with posterior laryngeal stenosis or tracheal stenosis also had a score above 10 points.

The scores of the different PROMs correlated with high significance. The Spearman’s rank correlation between S-EAT-10 in comparison to S-VHI-11, HADS-A, and HADS-D was 0.60 or higher (p ≤ 0.002). The correlation between S-VHI-11 and the two HADS subscales was 0.50 and 0.52 (p = 0.013 and p = 0.009, respectively). The subscales of the HADS questionnaire also correlated strongly (rho = 0.78; p < 0.001).

All 36 participants consumed drink and food exclusively by mouth at follow-up. On the specific questions concerning eating, drinking, and swallowing, three patients (8%) responded that they had subjective difficulties with eating, four (11%) with drinking, and five (14%) stated problems with swallowing.

### Judgment reliability

Ratings of pooling of secretion was equal in 94.1% and within one scale step in 100% of ratings (weighted κ = 0.92). For PAS, exact agreement between ratings was found in 95.6% and 97.8% of ratings within one scale step (weighted κ = 0.91). The intra-judge reliability was somewhat lower for ratings of residue. For ratings of residue in the vallecula, 63.7% were rated equally and 90.1% within one scale step (weighted κ = 0.63) (nine duplications [9.9%] were judged as not possible to assess in one of the two duplications). For ratings of residue in the pyriform sinuses, equal rating was present in 76.9% and 100% within one scale step (weighted κ = 0.69). Ratings of vocal fold movement were equal in 88.2% and ratings of vocal fold lesions were equal in 94.1% of the duplications.

## Discussion

This study indicates that a majority of patients with severe COVID-19 disease treated in the ICU regain normal or near-normal oropharyngeal swallowing function in longer term. However, symptoms and signs of dysphagia appear to persist in at least 10–20% of the patients at long-term follow-up performed 5–17 months after discharge from the ICU, depending on the method or instrument used for swallowing function assessment. In total, 22% of participants showed signs of penetration to the laryngeal vestibule which was not fully ejected (PAS ≥ 3) and 11% had silent aspiration (PAS = 8) on at least one occasion when swallowing thin liquid. Self-reported impairment of eating and swallowing (defined as a score of ≥ 3 on the S-EAT-10 questionnaire) was found in 40% of the responders. The S-EAT-10 score had low correlation in comparison to the results of the instrumental assessment of oropharyngeal swallowing with FEES, however.

This is, to our knowledge, the first long-term follow-up study of swallowing and laryngeal function in patients with severe COVID-19 treated in the ICU using fiberendoscopic examination. Previously, three studies have reported prevalence of nutritional and swallowing symptoms at follow-up after discharge from the hospital. In one study with 22 patients using a screening tool for dysphagia with four questions, the 4QT [38], 96% reported no nutritional problems one to two months after discharge [4]. In another study of 41 patients with severe COVID-19 who were tracheotomized and subsequently decannulated, 83% had total oral diet with no restrictions at 2-month follow-up [10]. Thirty percent had scores of ≥ 3 and 12% a score of ≥ 11 on the EAT-10 questionnaire in that study. In comparison, 40% of the responders had a score of ≥ 3 and 20% a score of > 11 on S-EAT-10 in the present study. In a third study of 205 COVID-19 patients [39] treated in general hospital wards, dysphagia (using a score of ≥ 2 on EAT-10 and a screening swallowing test to distinguish between patients with or without dysphagia) remained in 23% and 24% at 3 and 6 months, respectively, after discharge from the hospital. Interestingly, the S-EAT-10 score did not correlate with the findings from the FEES examination in our study. This suggests that the use of questionnaires alone is not enough for assessment of swallowing function in this patient group, but that instrumental assessment with, for example, FEES or videoradiography is essential.

In our previous study of swallowing function in COVID-19 patients treated in the ICU [9], a high incidence of pooling of secretion in the hypopharynx, silent aspiration, and residuals after swallowing was found, indicating neuromuscular weakness and/or decreased oropharyngeal and laryngeal sensation. The findings in the present study concerning PAS and residue after swallowing indicate that some individuals who were treated for severe COVID-19 in the ICU still may have remaining weakness in the base of tongue and the pharyngeal muscles several months after discharge. It is not possible to conclude whether this mainly is an effect of the long time in the ICU or if the COVID-19 infection is a complicating factor per se. It is well known that dysphagia is a common complication in ICU patients [40–43]. In a longitudinal follow-up study of dysphagia in intubated acute respiratory distress syndrome survivors [43], dysphagia symptoms (evaluated with a swallowing questionnaire) persisted beyond hospital discharge in one-third of patients. The data indicate that the longer the ICU length of stay, the slower the recovery from dysphagia. After 12 months, 25% of patients with an ICU length of stay of eight days still had not recovered. For patients with an ICU stay of 18 days, it took 24 months for 75% of patients to recover [43]. The fact that at least 20% of participants in the present study reported difficulties in eating and swallowing at follow-up indicates that dysphagia persists in this group of COVID-19 patients to a similar degree. There were no significant correlations between dysphagia signs and symptoms and the number of days in the ICU or in the hospital in the present study, however.

About half of the patients in the present study demonstrated abnormal findings in the larynx at the long-term follow-up. Impaired vocal fold movement was the most frequent finding, which was seen in 33% of patients. Contact granuloma or contact ulcer was found in 20% of participants. Two had visible tracheal stenosis and one patient had synechia in the posterior larynx. These findings are in concordance with previous studies of laryngeal characteristics in patients with severe COVID-19 disease. In a review of 393 patients [14], vocal fold dysmotility was reported in 65%, posterior glottic stenosis in 12%, and granuloma in 14% of patients examined with fiberendoscopy in the post-acute stage after COVID-19. In a retrospective study, Shah and collaborators [17] found vocal fold paresis/paralysis in 46%, structural vocal fold alterations in 21%, and laryngotracheal stenosis in 42% of intubated COVID-19 patients at follow-up at an average of 175 days from COVID-19 infection. Unilateral vocal cord palsy was noted in 8% and subglottic stenosis in 5% of patients who underwent tracheostomy due to COVID-19 at examination 2 months following discharge from the hospital in a study by Rouhani and colleagues [10]. The median VHI-10 score was 11 points in the study by Shah et al*.* [17] and 13% (5 of 38) had a VHI-10 score above 11 points in the study by Rouhani et al*.* [10]. In comparison, the median S-VHI-11 score was 5 points and 28% had a S-VHI-11 score above 12 points in the present study. The relatively high prevalence of abnormal findings in the larynx indicates that laryngeal examination should be included in the follow-up of patients treated for severe COVID-19 in the ICU.

It is yet to be determined whether the impairment of vocal fold motion in patients with severe COVID-19 disease is a direct cause of the treatment in the ICU or if other factors related to the COVID-19 infection also are involved. Laryngeal injury from endotracheal intubation is common in the ICU setting, at least in the short term. Brodsky et al*.* [44] reported a 21% prevalence of vocal fold immobility, a 31% prevalence of ulceration, and a 27% prevalence of granulomas/granulomatous tissue in a review of laryngeal sequelae after oral endotracheal intubation in critical care shortly after extubation in nine different studies. The laryngeal examinations were performed within 6 h to 2 weeks after extubation in these studies. Research examining long-term follow-up of laryngeal function after intubation and ICU treatment is lacking [45]. In a recent review, Kelly and co-authors [45] identified no long-term studies of persistent features of laryngeal injury in non-COVID-19 patients treated in the ICU. It has been suggested that post-viral vagal neuropathy may be a cause of vocal fold paresis in COVID-19 patients [46]. More research is needed in this context.

In addition to dysphagia and abnormal laryngeal findings, the PROM data in the present study indicate that about one-third of the responders had a symptom score indicating at least possible anxiety and one-fourth of patients a symptom score indicating at least possible depression at follow-up. The anxiety and depression scores appeared to correlate with subjective difficulties with eating, swallowing, and voice function. These findings are in line with previously reported data of long-term sequelae related to emotional and mental function after ICU treatment for severe COVID-19. One year after ICU treatment, 18% of COVID-19 patients had an anxiety score ≥ 8 points (corresponding to at least possible anxiety) and 18% a depression score of ≥ 8 points (corresponding to at least possible depression) on the HADS questionnaire in a study by Heesakkers et al*.* [47]. In another study [48] 46% had symptoms of anxiety and 34% symptoms of depression at 3–7 months post-hospital discharge among patients admitted to critical care due to severe COVID-19. A similar prevalence of anxiety and depression has been reported in non-covid ICU survivors after one year [49, 50].

### Limitations

The authors acknowledge several limitations in this study. The study cohort included a group of COVID-19 patients who accepted participation in the follow-up study, where only approximately half were evaluated with FEES during their ICU treatment. It is not possible to determine with certainty to what extent the study group is representative of the entire group of patients with severe COVID-19 treated in the ICU at our hospital, but comparison to a national cohort study of 1,649 Swedish COVID-19 patients treated in ICU from 2020–03-06 to 2020–08-23 [51], there were some possible differences. For example, the proportion of men was slightly higher in the present study (89% vs 75%), the mean age slightly higher (64 vs 60 years), the number of obese (BMI > 30) participants somewhat higher (47% vs 39%), and the mean length of stay in the ICU longer (24 vs 15 days) [51]. The prevalence of comordibities were also different with a higher proportion of patients with hypertension (64% vs 19%) and diabetes (47% vs 12%) in our study, but the proportion with cardiovascular diseases was lower (22% vs 33%). Only about 70% (25/36) of participants responded to the PROMs, which also is a limitation. There were no statistical differences regarding the rest of the variables between responders and non-responders, however. Thus, it appears possible to interpret the PROM data as representative for the whole group of participants.

## Conclusion

This is the first study using instrumental examination of swallowing function with FEES in long-term follow-up after treatment of severe COVID-19 disease in the ICU, to our knowledge. Although a majority of patients appear to regain normal swallowing function at follow-up up to more than one year after treatment for severe COVID-19 disease in the ICU, the results from this study indicate that symptoms and/or signs of dysphagia, abnormal laryngeal function, and anxiety and depression may remain in a substantial proportion of patients. This suggests that swallowing and laryngeal function, as well as emotional symptoms, should be followed up systematically over time in this patient group.

## Data Availability

The datasets generated during and analysed during the current study are not publicly available due to ethical reasons.
